# A more reliable species richness estimator based on the Gamma–Poisson model

**DOI:** 10.7717/peerj.14540

**Published:** 2023-01-06

**Authors:** Chun-Huo Chiu

**Affiliations:** Department of Agronomy, National Taiwan University, Taipei, Taiwan

**Keywords:** Richness, Good-Turing frequency formula, Parametric method, Gamma-Poisson model, Diversity

## Abstract

**Background:**

Accurately estimating the true richness of a target community is still a statistical challenge, particularly in highly diverse communities. Due to sampling limitations or limited resources, undetected species are present in many surveys and observed richness is an underestimate of true richness. In the literature, methods for estimating the undetected richness of a sample are generally divided into two categories: parametric and nonparametric estimators. Imposing no assumptions on species detection rates, nonparametric methods demonstrate robust statistical performance and are widely used in ecological studies. However, nonparametric estimators may seriously underestimate richness when species composition has a high degree of heterogeneity. Parametric approaches, which reduce the number of parameters by assuming that species-specific detection probabilities follow a given statistical distribution, use traditional statistical inference to calculate species richness estimates. When species detection rates meet the model assumption, the parametric approach could supply a nearly unbiased estimator. However, the infeasibility and inefficiency of solving maximum likelihood functions limit the application of parametric methods in ecological studies when the model assumption is violated, or the collected data is sparse.

**Method:**

To overcome these estimating challenges associated with parametric methods, an estimator employing the moment estimation method instead of the maximum likelihood estimation method is proposed to estimate parameters based on a Gamma-Poisson mixture model. Drawing on the concept of the Good-Turing frequency formula, the proposed estimator only uses the number of singletons, doubletons, and tripletons in a sample for undetected richness estimation.

**Results:**

The statistical behavior of the new estimator was evaluated by using real and simulated data sets from various species abundance models. Simulation results indicated that the new estimator reduces the bias presented in traditional nonparametric estimators, presents more robust statistical behavior compared to other parametric estimators, and provides confidence intervals with better coverage among the discussed estimators, especially in assemblages with high species composition heterogeneity.

## Introduction

Species richness is the most commonly used diversity index and a key metric in ecological research. Due to the rapid expansion of the human population and the disturbance induced by human activities that increasingly eliminates ecological habitats, an increasing number of species become extinct before even being discovered (Costello, May & Stork, 2013). Assessment and long-term monitoring of species diversity in a target area has become an urgent task for conservation biologists.

To collect and identify all species in a target area, researchers need to conduct a census of all species in the area. However, generating species inventories of a target area requires enormous investigation efforts and is often impractical due to resource limitations. Therefore, most biodiversity studies are based on the sampled data from the target area or assemblage. However, since species sampling data represent only a partial collection of the entire assemblage, it is hard to detect all species of the assemblage, especially when the sample size is small. Because the true number of species in an area equates to the number of species observed in the sample plus the number of species not appearing in the sample, using the recorded number of species in the sample as an estimator will lead to underestimating the true richness of the target area or assemblage.

In general, the number of undetected species in a sample depends on sampling effort and sample completeness (Hortal, Borges & Gaspar, 2006). Accurate estimation of the species richness in an area is still a statistical challenge, especially in a highly heterogeneous assemblage (Bunge, Willis & Walsh, 2014). Researchers in different disciplines have developed methods for estimating the number of species according to different sampling schemes or model assumptions (Bunge & Fitzpatrick, 1993; Chao & Chiu, 2012; Colwell & Coddington, 1994; Gotelli & Colwell, 2011; Lanumteang & Böhning, 2011; Norris & Pollock, 1998). The proposed estimators are generally classified as nonparametric or parametric. As nonparametric richness estimators do not impose model assumptions on species detection probability, they are more robust and more frequently used by ecologists or conservationists. Among the nonparametric approaches, the Chao1 lower bound estimator (Chao, 1984) and jackknife estimator (Burnham & Overton, 1978; Burnham & Overton, 1979) are the most commonly used methods. Based on the concept that rare species in a sample contain most of the information about undetected species, these nonparametric estimation methods use the number of species observed only once or twice in the sample to estimate the number of undetected species. However, the nonparametric species estimators often considerably underestimate the true number of species, particularly when the sample size is small or when the assemblage has a high degree of heterogeneity (Chao, 2005). Conversely, parametric methods treat the species detection rate as a random variable following a specific probability distribution. Under this assumption, estimating the number of species becomes a matter of estimating the parameters of the probability distribution, and traditional statistical inference approaches may be applied. Generally, a computationally expensive, iterative numerical algorithm is required to solve this parameter estimation problem by using the maximum likelihood method. When the distribution of the true species detection probability is similar to the hypothetical distribution, a parametric richness estimator provides a more accurate estimate. However, when the community is highly heterogeneous and the sample size is not sufficiently large, the parametric estimator frequently fails to converge or requires additional computing time, especially when the sampled data are sparse. Therefore, parametric methods are less frequently adopted to assess species diversity in ecological studies.

This study proposes a parametric estimation method in which the species detection probability is assumed to be a random variable following a probability distribution. In addition, the Good-Turing frequency formula (Good & Toulmin, 1956; Good, 1953) reveals that the rare species in a sample contain most of the information concerning undetected species. In this case, the moment method is used to estimate the parameters of the distribution instead of employing the time-consuming maximum likelihood method. Consequently, the proposed method overcomes the problems of statistical divergence and time-consuming parameter calculations encountered in applying the maximum likelihood method. Furthermore, similar to the nonparametric approach, the proposed richness estimator estimates the number of undetected species on the basis of only the sample’s rare species data (*i.e.,* the number of singletons, doubletons, and tripletons). Thus, fieldwork may be substantially reduced because researchers are only required to record the number of rare species, not the exact individual number of abundant species in the field.

In the following section, the hypothetical model of species composition and the theoretical framework of the proposed estimation method are detailed. Subsequently, in the simulation analysis section, the statistical performances (*e.g.*, bias, the estimated standard error (s.e.), and the coverage rate on a 95% confidence interval) of the proposed estimator are analyzed in various common species composition models. Across different real data sets, the proposed approach is analyzed and compared with commonly used estimators. In the final section, the findings of this research are discussed.

## Materials & Methods

For individual-based abundance data, the sampling unit is an individual and one individual is randomly sampled and identified at a time. Assume there are *S* species in the target area or assemblage, which is an unknown parameter. Let *X*_*i*_ be the number of individuals of the *i* th species observed in the sample. When the assemblage is sampled for a fixed period of time, *X*_*i*_ follows the Poisson model with discovery rate *λ*_*i*_, *i* = 1, 2, …, *S*. To simultaneously reduce the number of unknown parameters and consider the heterogeneity of the species detection rate, let *λ*_*i*_ be a random variable following a specific distribution. In ecological studies, a mixed Poisson model assumes that the count *X*_*i*_ follows a Poisson distribution *P*(*λ*_*i*_), where *λ*_1_, *λ*_2_, …, *λ*_*S*_ are independent and identically distributed random variables from a probability density function $g \left( \lambda \right) $ with a few parameters. Here, I presume $g \left( \lambda \right) $ is from a gamma distribution with two parameters (*α*, *β*), where *α* is a shape parameter and *β* is a scale parameter. When *α* = 1, $g \left( \lambda \right) $ is equal to the exponential distribution, which corresponds to the well-known broken-stick model. When *α* tends to infinity, $g \left( \lambda \right) $ will converge to the uniform distribution, which is identical to a homogeneous model in ecological studies. Therefore, the Poisson-Gamma model is a flexible model, and many estimators and richness estimators have been proposed based on this model assumption (Sanathanan, 1972; Sanathanan, 1977; Chao & Bunge, 2002; Lanumteang & Böhning, 2011).

On the basis of the Gamma-Poisson mixture model assumption, the marginal distribution of the species count in the sample can be expressed as follows:

(1) \begin{eqnarray*}{p}_{k}=P \left( {X}_{i}=k \right) =\int \nolimits \nolimits _{0}^{\infty } \frac{{\lambda }^{k}}{k{!}} {e}^{-\lambda } \frac{{\lambda }^{\alpha -1}{\beta }^{\alpha }}{\Gamma (\alpha )} {e}^{-\beta \lambda }d\lambda = \frac{\Gamma \left( \alpha +k \right) }{k{!}\Gamma \left( \alpha \right) } { \left( \frac{\beta }{\beta +1} \right) }^{\alpha }{ \left( \frac{1}{\beta +1} \right) }^{k},\nonumber\\\displaystyle \quad k=0,1,2,\ldots .\end{eqnarray*}

Let the species frequency count *f*_*k*_ denote the number of species observed exactly *k* times in the sample; that is ${f}_{k}={\mathop{\sum }\nolimits }_{i=1}^{S}I({X}_{i}=k),k=0,1,2,\ldots $ , where *I* (A) is an indicator function. *I*(*A*) is equal to 1 if event *A* occurs, and *I*(*A*) is equal to 0 otherwise. Thus, *f*_0_ is the number of undetected species in the sample, and ${S}_{\mathrm{obs}}={\mathop{\sum }\nolimits }_{k=1}^{n}{f}_{k}$ is the observed richness in the sample. In this case, the observed frequency count {*f*_*k*_:*k* ≥ 1} in the sample follows a multinomial distribution with total sum *S*_obs_ and cell probabilities $\{ \frac{{p}_{k}}{1-{p}_{0}} :k\geq 1\} $. Therefore, the likelihood function can be expressed as $L \left( \alpha ,\beta \right) = \frac{{S}_{\mathrm{obs}}{!}}{{\prod }_{k\geq 1}{f}_{k}{!}} {\prod }_{k\geq 1}{ \left( \frac{{p}_{k}}{1-{p}_{0}} \right) }^{{f}_{k}}$. The maximum likelihood estimator of *α* and *β* can then be obtained by using an iterative numerical procedure (Sanathanan, 1972; Sanathanan, 1977). The mean detection probability of the unobserved species can be obtained using ${\hat {p}}_{0}={ \left( \frac{\hat {\beta }}{\hat {\beta }+1} \right) }^{\hat {\alpha }}$. Following the Horvitz-Thompson theory, we have $S=E \left[ \frac{{\mathop{\sum }\nolimits }_{i=1}^{S}I \left( {X}_{i}\gt 0 \right) }{1-{p}_{0}} \right] $. Then, the ML estimator of richness can be obtained as ${\hat {S}}_{MLE}= \frac{{S}_{\mathrm{obs}}}{1-{\hat {p}}_{0}} $.

Chao & Bunge (2002) developed an alternative estimation method based on a Gamma–Poisson mixture model. The researchers showed $\hat {\theta }=1- \frac{{f}_{1}{\mathop{\sum }\nolimits }_{i=1}^{{S}_{\mathrm{obs}}}{X}_{i}^{2}}{n} $ converges to $P \left( {X}_{i}\geq 2 \right) $ in probability, which is equivalent to the assertion that $S\times \hat {\theta }$ converges to ∑_*k*≥2_*f*_*k*_ in probability; they subsequently proposed another richness estimator expressed as:

(2) \begin{eqnarray*}{\hat {S}}_{CB}= \frac{1}{\hat {\theta }} \sum _{k\geq 2}{f}_{k}.\end{eqnarray*}

Compared with the traditional maximum likelihood estimator, ${\hat {S}}_{CB}$ ispresented as a closed formula which is instantaneous to compute. However, these two parametric approaches are less commonly used in ecological studies because of the divergence problem which can arise when sparsely sampled data is applied.

As indicated by the estimator formulas, the two parametric estimators use all observed species data in the sample, including abundant and rare species, to estimate unseen richness. However, the Good–Turing frequency formula indicates that observed rare species contain most of the information about unobserved species. According to this concept, abundant species in the sample mostly do not contain information about the undetected richness and may generate nuisance statistics in estimations of undetected species richness, resulting in high variance and instability. Therefore, abundant species in the sample usually are excluded from richness estimation to obtain more robust estimators (Chao, 1984; Chao, 1987; Chao & Yang, 1993; Chao et al., 2000). Herein, a new richness estimator is derived that employs a simple moment approach to sampled rare species data. According to the Gamma-Poisson mixture model, the expected species frequency count in the sample can be expressed as follows:

\begin{eqnarray*}E \left[ {f}_{k} \right] =S\times {p}_{k}=S \frac{\Gamma \left( \alpha +k \right) }{k{!}\Gamma \left( \alpha \right) } { \left( \frac{\beta }{\beta +1} \right) }^{\alpha }{ \left( \frac{1}{\beta +1} \right) }^{k}. \end{eqnarray*}

The unseen richness as well as the numbers of singletons, doubletons, and tripletons can be derived as follows:

(3a) \begin{eqnarray*}E \left[ {f}_{0} \right] & =S{ \left( \frac{\beta }{\beta +1} \right) }^{\alpha },\end{eqnarray*}

(3b) \begin{eqnarray*}E \left[ {f}_{1} \right] & =S \frac{\alpha }{\beta +1} { \left( \frac{\beta }{\beta +1} \right) }^{\alpha },\end{eqnarray*}

(3c) \begin{eqnarray*}E \left[ {f}_{2} \right] & =S \frac{\alpha \left( \alpha +1 \right) }{2{ \left( \beta +1 \right) }^{2}} { \left( \frac{\beta }{\beta +1} \right) }^{\alpha },\end{eqnarray*}

(3d) \begin{eqnarray*}E \left[ {f}_{3} \right] & =S \frac{\alpha (\alpha +1)(\alpha +2)}{6{ \left( \beta +1 \right) }^{3}} { \left( \frac{\beta }{\beta +1} \right) }^{\alpha }.\end{eqnarray*}

According to Eqs. (3a), (3b), (3c) and (3d), the following equalities can be obtained as:

(4a) \begin{eqnarray*} \frac{E \left[ {f}_{0} \right] }{E \left[ {f}_{1} \right] } & = \frac{\beta +1}{\alpha } ,\end{eqnarray*}

(4b) \begin{eqnarray*} \frac{E \left[ {f}_{1} \right] }{E \left[ {f}_{2} \right] } & = \frac{2 \left( \beta +1 \right) }{\alpha +1} ,\end{eqnarray*}

(4c) \begin{eqnarray*} \frac{E \left[ {f}_{2} \right] }{E \left[ {f}_{3} \right] } & = \frac{3 \left( \beta +1 \right) }{\alpha +2} .\end{eqnarray*}

According to Eqs. (4b) and (4c), the scale parameter *α* in the mixed Poisson model is identical to the following:

\begin{eqnarray*}\alpha = \frac{4E{ \left[ {f}_{2} \right] }^{2}-3E[{f}_{1}]E[{f}_{3}]}{3E \left[ {f}_{1} \right] E \left[ {f}_{3} \right] -2E{ \left[ {f}_{2} \right] }^{2}} . \end{eqnarray*}

Then, the estimator of *α* can be obtained by: $\hat {\alpha }= \frac{4{f}_{2}^{2}-3{f}_{1}{f}_{3}}{3{f}_{1}{f}_{3}-2{f}_{2}^{2}} $. To ensure $\hat {\alpha }\gt 0$, 3*f*_1_*f*_3_ should be within the range of $ \left( 2{f}_{2}^{2},4{f}_{2}^{2} \right) $. According to Eqs. (4a) and (4b), the expected unseen richness can also be presented as follows:

\begin{eqnarray*}E[{f}_{0}]= \frac{E{ \left[ {f}_{1} \right] }^{2}}{2E \left[ {f}_{2} \right] } \left( 1+ \frac{1}{\alpha } \right) . \end{eqnarray*}

Since the Cauchy–Schwarz inequality could show $ \frac{E{ \left[ {f}_{1} \right] }^{2}}{2E \left[ {f}_{2} \right] } $ as a lower bound of *E*[*f*_0_] (Chao, 1984), it is implied that $ \frac{E{ \left[ {f}_{1} \right] }^{2}}{2E[{f}_{2}]} \left( \frac{1}{\alpha } \right) $ isthe bias of $ \frac{{f}_{1}^{2}}{2{f}_{2}} $. Therefore, $ \frac{{f}_{1}^{2}}{2{f}_{2}} \left( 1+ \frac{1}{\hat {\alpha }} \right) $ is an unbiased estimator of undetected richness in the Gamma-Poisson mixture model. However, like all parametric estimators, this unbiased estimator is also unreliable when data is sparse. To obtain a more stable estimator, this unbiased estimator is modified to propose a new estimator. By the Cauchy–Schwarz inequality, the following inequality is held:

\begin{eqnarray*}\sum _{i=1}^{S}{p}_{i}{ \left( 1-{p}_{i} \right) }^{n-1}\sum _{i=1}^{S}{p}_{i}^{3}{ \left( 1-{p}_{i} \right) }^{n-3}\geq { \left( \sum _{i=1}^{S}{p}_{i}^{2}{ \left( 1-{p}_{i} \right) }^{n-2} \right) }^{2}, \end{eqnarray*}

which is approximately equal to the inequality $2E{ \left[ {f}_{2} \right] }^{2}\leq 3E[{f}_{1}]E \left[ {f}_{3} \right] $ when sample size is sufficiently large. The lower bound of $ \frac{E{ \left[ {f}_{1} \right] }^{2}}{2E[{f}_{2}]} \frac{1}{\alpha } $ can be obtained as:

\begin{eqnarray*} \frac{E{ \left[ {f}_{1} \right] }^{2}}{2E \left[ {f}_{2} \right] } \frac{1}{\alpha } = \frac{E{ \left[ {f}_{1} \right] }^{2}}{2E \left[ {f}_{2} \right] } \left( \frac{3E \left[ {f}_{1} \right] E \left[ {f}_{3} \right] -2E{ \left[ {f}_{2} \right] }^{2}}{4E{ \left[ {f}_{2} \right] }^{2}-3E \left[ {f}_{1} \right] E \left[ {f}_{3} \right] } \right) \geq \frac{E{ \left[ {f}_{1} \right] }^{2}}{2E \left[ {f}_{2} \right] } \left( 1- \frac{2E{ \left[ {f}_{2} \right] }^{2}}{3E \left[ {f}_{1} \right] E \left[ {f}_{3} \right] } \right) . \end{eqnarray*}

To obtain a more stable (less RMSE) estimator of unseen richness and ensure $\hat {\alpha }\gt 0$, the proposed richness estimator is as follows:

(5) \begin{eqnarray*}{\hat {S}}_{GP}={S}_{\mathrm{obs}}+{\hat {f}}_{0.Chao1} \left( 2-{ \left( \frac{2{f}_{2}^{2}}{3{f}_{1}{f}_{3}} \right) }^{-} \right) ,\end{eqnarray*}

where ${\hat {f}}_{0.Chao1}= \left\{ {\scriptsize \begin{array}{@{}ll@{}} \displaystyle {f}_{1}^{2}/2{f}_{2} &\displaystyle if{f}_{2}\gt 0\\ \displaystyle {f}_{1}({f}_{1}-1)/2 &\displaystyle if{f}_{2}=0 \end{array}} \right. $ and the expression ${ \left( A \right) }^{-}$ equals $\max ( \frac{1}{2} ,A)$ if *A* < 1 and 1 if *A* ≥ 1. Here, *f*_3_ (or *f*_1_) is replaced by 1 when *f*_3_ = 0 (or *f*_1_ = 0) to ensure that Eq. (5) is always well-defined. Since ${f}_{1}^{2}/(2{f}_{2})$ is the lower bound estimator of unseen richness (Chao, 1984), the newly proposed estimation method can be treated as a bias-corrected estimator of Chao1 under the Gamma-Poisson mixture model. Furthermore, in Appendix S1A, it is shown that the newly proposed estimator can also be directly derived by correcting the bias of Chao1 based on the Good-Turing frequency formula without any model assumptions. Notably, when the homogeneity of species composition is met, we have $2E{ \left[ {f}_{2} \right] }^{2}=3E \left[ {f}_{1} \right] E \left[ {f}_{3} \right] $ and $E{ \left[ {f}_{1} \right] }^{2}=2E \left[ {f}_{0} \right] E \left[ {f}_{2} \right] $. This implies that the proposed estimator will be approximately identical to the Chao1 lower bound estimator and both are unbiased estimators for a homogeneous model (proved in Appendix S2B).

Under the assumption of the Gamma-Poisson mixture model, the marginal distribution of species count (Eq. (1)) is identical to a negative binomial distribution. Lanumteang & Böhning (2011) reformulated the parameters by using the Taylor expansion and derived a richness estimator expressed as:

(6) \begin{eqnarray*}{\hat {S}}_{LB}={S}_{\mathrm{obs}}+ \frac{{f}_{1}^{2}}{2{f}_{2}} \frac{3{f}_{1}{f}_{3}}{2{f}_{2}^{2}} .\end{eqnarray*}

Since $3E \left[ {f}_{1} \right] E \left[ {f}_{3} \right] \geq 2E{ \left[ {f}_{2} \right] }^{2}$ is held by the Cauchy–Schwarz inequality, ${\hat {S}}_{LB}$ was interpreted as a bias-corrected Chao1 estimator (Lanumteang & Böhning, 2011).

Here, ${\hat {S}}_{LB}$ and ${\hat {S}}_{GP}$, both interpreted as bias-corrected Chao1 estimators, were derived by using the moment method based on the assumption of the Gamma-Poisson mixture model and using the numbers of singletons, doubletons, and tripletons species to estimate the unseen richness in the sample. However, ${\hat {S}}_{LB}$ and ${\hat {S}}_{GP}$ have quite different formulaic expressions due to differences in how they adjust to correct the negative bias of Chao1; their statistical performances are also quite different as will be presented in the following simulation section.

Using an asymptotic approach, we can derive the estimators of variance of the discussed richness estimators that use rare species frequency counts to estimate undetected richness, based on the assumption that $ \left( {f}_{0},{f}_{1},\ldots ,{f}_{n} \right) $ approximately follows a multinomial distribution with total size *S* and cell probabilities $( \frac{E[{f}_{0}]}{S} , \frac{E[{f}_{1}]}{S} ,\ldots , \frac{E \left[ {f}_{n} \right] }{S} )$ (Chao & Lee, 1992; Chao & Yang, 1993). Consequently, we have the following:

$\widehat{\mathrm{var}}(\hat {S})\approx {\mathop{\sum }\nolimits }_{i=1}^{n}{\mathop{\sum }\nolimits }_{j=1}^{n} \frac{\partial \hat {S}}{\partial {f}_{i}} \frac{\partial \hat {S}}{\partial {f}_{j}} \widehat{\mathrm{cov}} \left( {f}_{i},{f}_{j} \right) $, where $\widehat{\mathrm{cov}} \left( {f}_{i},{f}_{j} \right) = \left\{ {\scriptsize \begin{array}{@{}ll@{}} \displaystyle {f}_{i}(1-{f}_{i}/\hat {S}) &\displaystyle ifi=j\\ \displaystyle -{f}_{i}{f}_{j}/\hat {S} &\displaystyle ifi\not = j \end{array}} \right. $.

To derive the 95% confidence interval (95% CI) of species richness and to ensure that the lower bound of the 95% CI of species richness is larger than the observed richness, assume $\hat {S}-{S}_{\mathrm{obs}}$ follows a log-normal distribution (Chiu et al., 2014). Then the 95% CI of species richness is obtained as $[{S}_{\mathrm{obs}}+ \frac{\hat {S}-{S}_{\mathrm{obs}}}{R} ,{S}_{\mathrm{obs}}+(\hat {S}-{S}_{\mathrm{obs}})R]$, where $R=\mathrm{exp} \left\{ 1.96{ \left[ \log \left( 1+ \frac{\mathrm{V ar} \left( \hat {S} \right) }{{ \left( \hat {S}-{S}_{\mathrm{obs}} \right) }^{2}} \right) \right] }^{ \frac{1}{2} } \right\} $.

According to this derivation, the proposed richness estimator has the following properties: (i) Instead of requiring all sample data as in existing parametric approaches, the proposed estimator uses the number of singletons, doubletons, and tripletons to estimate the unseen richness. (ii) Inefficiency and divergence problems encountered in solving the maximum likelihood function of parameters through iterative calculation methods in sparse data are avoided. (iii) The new estimator is asymptotically unbiased when sample size *n* is sufficiently large. (iv) The proposed estimator provides a lower bound estimator under the species composition assumption of the Gamma–Poisson mixture model, which is a flexible ecological model that incorporates the broken-stick model and the homogeneous model. (v) The newly proposed estimator can be directly derived by correcting the bias of Chao1 based on the Good-Turing frequency formula without any model assumptions. (vi) The new richness estimator can be interpreted as a bias-corrected Chao1 estimator and stay unbiased when species detection probability is homogeneous.

### Simulation study and results

We investigated the performance of the proposed estimator $({\hat {S}}_{GP})$ and compared it with that of the previously described estimators, namely two nonparametric approaches (the Chao1 lower bound estimator, denoted as ${\hat {S}}_{Chao1}$, and the first-order jackknife estimator, denoted as ${\hat {S}}_{Jack1}$) and two parametric approaches (${\hat {S}}_{CB}$ and ${\hat {S}}_{LB}$) derived under the Gamma-Poisson mixture model. Herein, other parametric approaches are excluded due to the divergence problem in calculating the maximum likelihood estimation (MLE) of parameters which makes it difficult to fairly compare with other estimators. The simulation study was conducted using two assemblage settings: one of the settings involved calculating species composition from seven models, and the other involved treating three data sets as the entire assemblage.

#### Species composition generated from the theoretical abundance model

The simulation results were obtained from seven commonly used ecological species abundance models. The number of species in each model was set to *S* = 1,000. The species detection probabilities or species relative abundances $ \left( {p}_{1},{p}_{2},\ldots ,{p}_{S} \right) =(c{a}_{1},c{a}_{2},\ldots ,c{a}_{S})$ in each model are provided subsequently, where *c* is a normalizing constant such that ${\mathop{\sum }\nolimits }_{i=1}^{S}{p}_{i}=1$. We also present the coefficient of variation (CV) of (*p*_1_, *p*_2_, …, *p*_*S*_) to indicate the degree of heterogeneity of $ \left( {p}_{1},{p}_{2},\ldots ,{p}_{S} \right) $.

Model 1, homogeneous model (CV = 0): with *p*_*i*_ = 1/*S*, *i* = 1, 2, …, *S*. This model has no heterogeneity among species detection probabilities.

Model 2, random uniform model (CV = 0.53): with *p*_*i*_ = *ca*_*i*_, *i* = 1, 2, …, *S*, where

$ \left( {a}_{1},{a}_{2},\ldots ,{a}_{S} \right) $ isa random sample from the uniform distribution.

Model 3, negative binomial model (CV = 0.74): with *p*_*i*_ = *ca*_*i*_, *i* = 1, 2, …, *S*, where

$ \left( {a}_{1},{a}_{2},\ldots ,{a}_{S} \right) $ isa random sample from the negative binomial distribution with a mean of 98 and a variance of 4,900.

Model 4, broken-stick model (CV = 0.97): with *p*_*i*_ = *ca*_*i*_, *i* = 1, 2, …, *S*, where $ \left( {a}_{1},{a}_{2},\ldots ,{a}_{S} \right) $ is a random sample from the exponential distribution with parameter 1. This model is commonly used in the literature and also equivalent to the Dirichlet distribution.

Model 5, log-normal model (CV = 1.56): with *p*_*i*_ = *ca*_*i*_, *i* = 1, 2, …, *S*, where $ \left( {a}_{1},{a}_{2},\ldots ,{a}_{S} \right) $ is a random sample from the log-normal distribution with parameters 0 and 1.

Model 6, Zipf–Mandelbrot model (CV = 1.88): with ${p}_{i}= \frac{c}{i+10} ,i=1,2,\ldots ,S$.

Model 7, power decay model (CV = 4): with ${p}_{i}= \frac{c}{{i}^{0.9}} ,i=1,2,\ldots ,S$.

The CV values in these seven models ranged from 0 to 4 and covered the majority of practical scenarios in real cases. Four different sample sizes were considered: 1,000, 2,000, 4,000 and 8,000. Therefore, a total of 28 model-size combinational scenarios were produced. For each model and sample size, 1,000 simulated data sets were generated, and the following estimators were used to derive estimations:

(i) The Chao1 estimator (${S}_{\mathrm{obs}}+ \frac{{f}_{1}^{2}}{2{f}_{2}} $): using the number of singletons and doubletons to estimate the number of unseen species.
(ii) The first-order jackknife estimator (*S*_obs_ + *f*_1_): using the number of singletons to estimate the number of undetected species.
(iii) The parametric estimator proposed by Chao & Bunge (2002); see Eq. (2).
(iv) The parametric estimator proposed by Lanumteang & Böhning (2011); see Eq. (6).
(v) The newly proposed richness estimator; see Eq. (5).

For each estimator, the estimate and the corresponding estimated s.e. were averaged over the 1,000 simulated data sets to derive the average estimate and the average estimated s.e.. The sample s.e. and root-mean-square error (RMSE) over the 1,000 estimates were also obtained. The percentage of data sets in which the 95% confidence intervals covered the true value is presented in Tables 1–7. The average richness observed in the 1,000 samples is also listed in the Tables.

**Table 1:** Comparison of five richness estimators based on 1,000 simulation data sets under a homogeneous model with *S* = 1,000 and CV = 0. The five estimators are: the Chao1 estimator (Chao, 1984) denoted as ${\hat {S}}_{\mathrm{Chao1}}$, the first-order Jackknife estimator (Burnham & Overton, 1978) denoted as ${\hat {S}}_{\mathrm{Jack1}}$, the estimator proposed by Chao & Bunge (2002) denoted as ${\hat {S}}_{\mathrm{CB}}$, the estimator proposed by Lanumteang & Böhning (2011) denoted as ${\hat {S}}_{\mathrm{LB}}$, and the newly proposed estimator denoted as ${\hat {S}}_{\mathrm{GP}}$.

Size *n* (Observed richness)	Estimator	Average estimate	Bias	Sample s.e.	Average estimated s.e.	Sample RMSE	95% CI coverage rate
1,000 (633.0)	${\hat {S}}_{\mathrm{Chao1}}$	1,006.4	6.4	51.5	51.4	51.9	0.94^a^
	${\hat {S}}_{\mathrm{Jack1}}$	1,002.2	2.2^a^	24.2	27.2	24.3^a^	0.97
	${\hat {S}}_{\mathrm{CB}}$	1,009	9	72.1	106.5	72.7	0.99
	${\hat {S}}_{\mathrm{LB}}$	1,019.9	19.9	122.4	118	124.0	0.93
	${\hat {S}}_{\mathrm{GP}}$	1,025.3	25.3	84.6	78.2	88.3	0.92
2,000 (864.4)	${\hat {S}}_{\mathrm{Chao1}}$	1,000.5	0.5	22.8	21.9	22.8	0.94^a^
	${\hat {S}}_{\mathrm{Jack1}}$	1,134.9	134.9	20.5	19.7	136.4	0
	${\hat {S}}_{\mathrm{CB}}$	1,000.2	0.2^a^	22	24.1	22^a^	0.96
	${\hat {S}}_{\mathrm{LB}}$	1,007.8	7.8	41.7	39.5	42.4	0.93
	${\hat {S}}_{\mathrm{GP}}$	1,006.4	6.4	32	30.4	32.6	0.93
4,000 (981.8)	${\hat {S}}_{\mathrm{Chao1}}$	1,000.4	0.4^a^	6	6.3	6	0.94
	${\hat {S}}_{\mathrm{Jack1}}$	1,055	55	8.7	9.4	55.7	0
	${\hat {S}}_{\mathrm{CB}}$	999.4	−0.6	5	5.3	5^a^	0.95^a^
	${\hat {S}}_{\mathrm{LB}}$	1,001.6	1.6	9.5	9.7	9.6	0.95^a^
	${\hat {S}}_{\mathrm{GP}}$	1,000.8	0.8	7.7	8.3	7.7	0.94
8,000 (999.7)	${\hat {S}}_{\mathrm{Chao1}}$	1,000.2	0.2	0.8	0.8	0.8	0.94^a^
	${\hat {S}}_{\mathrm{Jack1}}$	1,002.4	2.4	1.7	1.6	2.9	0.86
	${\hat {S}}_{\mathrm{CB}}$	999.9	−0.1^a^	0.6	0.6	0.6^a^	0.84
	${\hat {S}}_{\mathrm{LB}}$	1,001.1	1.1	4.1	2.9	4.2	0.84
	${\hat {S}}_{\mathrm{GP}}$	1,000.3	0.3	1.2	1.9	1.2	0.94^a^

**Notes.**

a denotes the smallest bias, smallest RMSE, and figure closest to 95% coverage.

**Table 2:** Comparison of five richness estimators based on 1,000 simulation data sets under a random uniform model with *S* = 1,000 and CV=0.56. See Table 1 for the notations of the estimators.

Size *n* (Observed richness)	Estimator	Average estimate	Bias	Sample s.e.	Average estimated s.e.	Sample RMSE	95% CI coverage rate
1,000 (572.3)	${\hat {S}}_{\mathrm{Chao1}}$	858.1	−141.9	44.1	43.2	148.6	0.2
	${\hat {S}}_{\mathrm{Jack1}}$	875.3	−124.7	23.6	24.2	126.9	0
	${\hat {S}}_{\mathrm{CB}}$	905.6	−94.4	67.4	86.9	115.9	0.76
	${\hat {S}}_{\mathrm{LB}}$	936.3	−63.7^a^	124	116.7	139.2	0.8
	${\hat {S}}_{\mathrm{GP}}$	922.1	−77.9	83.8	80.9	114.4^a^	0.84^a^
2,000 (762.7)	${\hat {S}}_{\mathrm{Chao1}}$	903.9	−96.1	25.4	24.2	99.4	0.1
	${\hat {S}}_{\mathrm{Jack1}}$	995.8	−4.2^a^	20	19.1	20.4^a^	0.93^a^
	${\hat {S}}_{\mathrm{CB}}$	916.9	−83.1	25.9	27	87.1	0.17
	${\hat {S}}_{\mathrm{LB}}$	956.7	−43.3	63.8	60.2	77.1	0.77
	${\hat {S}}_{\mathrm{GP}}$	942.3	−57.7	44	43	72.2	0.87
4,000 (886.1)	${\hat {S}}_{\mathrm{Chao1}}$	947.6	−52.4	15.7	14.5	54.7	0.19
	${\hat {S}}_{\mathrm{Jack1}}$	1,011.2	11.2^a^	14.8	13.5	18.5^a^	0.86
	${\hat {S}}_{\mathrm{CB}}$	938.4	−61.6	12	11.4	62.8	0
	${\hat {S}}_{\mathrm{LB}}$	977.3	−22.7	38.5	36.3	44.7	0.77
	${\hat {S}}_{\mathrm{GP}}$	970.7	−29.3	25.3	24.3	38.7	0.89^a^
8,000 (947.9)	${\hat {S}}_{\mathrm{Chao1}}$	975.7	−24.3	10.2	9.6	26.4	0.51
	${\hat {S}}_{\mathrm{Jack1}}$	1,006	6^a^	9.9	9.2	11.6^a^	0.88
	${\hat {S}}_{\mathrm{CB}}$	965.3	−34.7	7	6.7	35.4	0
	${\hat {S}}_{\mathrm{LB}}$	993.5	−6.5	28.2	26.4	28.9	0.79
	${\hat {S}}_{\mathrm{GP}}$	987.5	−12.5	15.5	15.5	19.9	0.92^a^

**Notes.**

a denotes the smallest bias, the smallest RMSE, and figure closest to 95% coverage.

**Table 3:** Comparison of five richness estimators based on 1,000 simulation data sets under a negative binomial model with *S* = 1,000 and CV = 0.74. See Table 1 for the notations of the estimators.

Size *n* (Observed richness)	Estimator	Average estimate	Bias	Sample s.e.	Average estimated s.e.	Sample RMSE	95% CI coverage rate
1,000 (554.8)	${\hat {S}}_{\mathrm{Chao1}}$	846.9	−153.1	47.2	45.9	160.1	0.19
	${\hat {S}}_{\mathrm{Jack1}}$	843	−157	23.9	24.2	158.8	0
	${\hat {S}}_{\mathrm{CB}}$	1,016.4	16.4^a^	121.5	128.4	122.4	0.94^a^
	${\hat {S}}_{\mathrm{LB}}$	966.6	−33.4	137.3	138.5	141.2	0.87
	${\hat {S}}_{\mathrm{GP}}$	931.6	−68.4	89.9	89.8	113.0^a^	0.87
2,000 (750.5)	${\hat {S}}_{\mathrm{Chao1}}$	916.7	−83.3	30.1	28.3	88.6	0.28
	${\hat {S}}_{\mathrm{Jack1}}$	996.1	−3.9^a^	21.9	20.4	22.2^a^	0.92^a^
	${\hat {S}}_{\mathrm{CB}}$	984.8	−15.2	40.8	40	43.5	0.9
	${\hat {S}}_{\mathrm{LB}}$	982	−18	78.5	73.6	80.5	0.87
	${\hat {S}}_{\mathrm{GP}}$	966.2	−33.8	52.3	51.6	62.3	0.91
4,000 (890.8)	${\hat {S}}_{\mathrm{Chao1}}$	961.5	−38.5	17.3	16.4	42.2	0.5
	${\hat {S}}_{\mathrm{Jack1}}$	1,035.8	35.8	15.8	14.9	39.1	0.31
	${\hat {S}}_{\mathrm{CB}}$	966.2	−33.8	15.1	14.9	37.1	0.4
	${\hat {S}}_{\mathrm{LB}}$	991.2	−8.8^a^	40.5	39	41.4	0.88
	${\hat {S}}_{\mathrm{GP}}$	986.3	−13.7	28.2	27.9	31.4^a^	0.92^a^
8,000 (960.7)	${\hat {S}}_{\mathrm{Chao1}}$	984.5	−15.5	9.7	9.1	18.3	0.72
	${\hat {S}}_{\mathrm{Jack1}}$	1,021.6	21.6	10	9.5	23.8	0.37
	${\hat {S}}_{\mathrm{CB}}$	977.5	−22.5	7	6.8	23.6	0.12
	${\hat {S}}_{\mathrm{LB}}$	997	−3^a^	23	21.3	23.2	0.86
	${\hat {S}}_{\mathrm{GP}}$	993.8	−6.2	14.7	14.3	16.0^a^	0.93^a^

**Notes.**

a denotes the smallest bias, smallest RMSE, and figure closest to 95% coverage.

**Table 4:** Comparison of five richness estimators based on 1,000 simulation data sets under a broken-stick model with *S* = 1,000 and CV = 1.01. See Table 1 for the notations of the estimators.

Size *n* (Observed richness)	Estimator	Average estimate	Bias	Sample s.e.	Average estimated s.e.	Sample RMSE	95% CI coverage rate
1,000 (499.8)	${\hat {S}}_{\mathrm{Chao1}}$	755	-245	42.2	42.7	248.6	0
	${\hat {S}}_{\mathrm{Jack1}}$	750.1	−249.9	22.6	22.4	250.9	0
	${\hat {S}}_{\mathrm{CB}}$	991.7	−8.3^a^	130.1	139.2	130.2^a^	0.92^a^
	${\hat {S}}_{\mathrm{LB}}$	907.1	−92.9	143.6	145.6	170.9	0.77
	${\hat {S}}_{\mathrm{GP}}$	861.7	−159.3	80.2	81.6	168.6	0.73
2,000 (666.5)	${\hat {S}}_{\mathrm{Chao1}}$	832	−168	31.9	29.1	171	0.01
	${\hat {S}}_{\mathrm{Jack1}}$	887.5	−112.5	22.1	19.5	114.7	0
	${\hat {S}}_{\mathrm{CB}}$	909.7	−90.3	44.4	43.2	100.6^a^	0.43
	${\hat {S}}_{\mathrm{LB}}$	923.7	−76.3^a^	97.5	87.2	123.8	0.67
	${\hat {S}}_{\mathrm{GP}}$	893.8	−114.2	56	53.3	120.1	0.77^a^
4,000 (799.6)	${\hat {S}}_{\mathrm{Chao1}}$	901.1	−98.9	22.3	21	101.3	0.05
	${\hat {S}}_{\mathrm{Jack1}}$	959.4	−40.6	17.3	16	44.1^a^	0.31
	${\hat {S}}_{\mathrm{CB}}$	906.3	−93.7	19.8	19.9	95.7	0.02
	${\hat {S}}_{\mathrm{LB}}$	961	−39^a^	66.2	60.9	76.7	0.75
	${\hat {S}}_{\mathrm{GP}}$	943.2	−56.8	37.8	37	68.2	0.79^a^
8,000 (889.7)	${\hat {S}}_{\mathrm{Chao1}}$	948.1	−51.9	16.6	15.3	54.4	0.24
	${\hat {S}}_{\mathrm{Jack1}}$	989.6	−10.4^a^	13.9	12.5	17.4^a^	0.83
	${\hat {S}}_{\mathrm{CB}}$	937.6	−62.4	12.5	12.1	63.6	0.01
	${\hat {S}}_{\mathrm{LB}}$	985.1	−14.9	46.8	44.4	49	0.81
	${\hat {S}}_{\mathrm{GP}}$	973.9	−26.1	26.9	25.9	37.5	0.87^a^

**Notes.**

a denotes the smallest bias, smallest RMSE, and figure closest to 95% coverage.

**Table 5:** Comparison of five richness estimators based on 1,000 simulation data sets under a log-normal model with *S* = 1,000 and CV = 1.35. See Table 1 for the notations of the estimators.

Size *n* (Observed richness)	Estimator	Average estimate	Bias	Sample s.e.	Average estimated s.e.	Sample RMSE	95% CI coverage rate
1,000 (481.1)	${\hat {S}}_{\mathrm{Chao1}}$	783.4	−216.6	51.6	50.6	222.7	0.07
	${\hat {S}}_{\mathrm{Jack1}}$	738.2	−261.8	23	23.5	262.8	0
	${\hat {S}}_{\mathrm{CB}}$	1,896	896	1,285.1	1,429.3	1,565.6	1
	${\hat {S}}_{\mathrm{LB}}$	982.8	−17.2^a^	192.2	187.6	192.8	0.85
	${\hat {S}}_{\mathrm{GP}}$	907.3	−92.7	99.1	96.9	135.7^a^	0.87^a^
2,000 (661.1)	${\hat {S}}_{\mathrm{Chao1}}$	874.5	−125.5	35.9	35.2	130.6	0.16
	${\hat {S}}_{\mathrm{Jack1}}$	913.9	−86.1	22.3	21.5	89	0.04
	${\hat {S}}_{\mathrm{CB}}$	1,067.3	67.3	80	79.5	104.4	0.96^a^
	${\hat {S}}_{\mathrm{LB}}$	991.4	−8.6^a^	114.6	109.4	114.8	0.9
	${\hat {S}}_{\mathrm{GP}}$	951.9	−48.1	66.6	66.1	82.2^a^	0.91
4,000 (818.2)	${\hat {S}}_{\mathrm{Chao1}}$	940.9	−59.1	24.4	23.1	64	0.44
	${\hat {S}}_{\mathrm{Jack1}}$	1,011.1	11.1	19	17.6	22^a^	0.89
	${\hat {S}}_{\mathrm{CB}}$	973.7	−26.3	26.5	26.5	37.3	0.77
	${\hat {S}}_{\mathrm{LB}}$	995.1	−4.9^a^	62.1	61.1	62.3	0.89
	${\hat {S}}_{\mathrm{GP}}$	979.4	−20.6	41.7	41.2	46.5	0.92^a^
8,000 (923.1)	${\hat {S}}_{\mathrm{Chao1}}$	978.9	−21.1	14.2	14	25.4	0.77
	${\hat {S}}_{\mathrm{Jack1}}$	1,034.2	34.2	13.4	12.8	36.7	0.22
	${\hat {S}}_{\mathrm{CB}}$	976.9	−23.1	12	12.1	26	0.49
	${\hat {S}}_{\mathrm{LB}}$	1,000.9	0.9^a^	32.8	33.2	32.8	0.91
	${\hat {S}}_{\mathrm{GP}}$	995.2	−4.8	22.7	23.2	23.2^a^	0.94^a^

**Notes.**

a denotes the smallest bias, smallest RMSE, and figure closest to 95% coverage.

**Table 6:** Comparison of five richness estimators based on 1,000 simulation data sets under a Zipf–Mandelbrot model *p*_*i*_ ∼ *C*/(*i* + 10) with *S* = 1,000 and CV = 1.87. See Table 1 for the notations of the estimators.

Size *n* (Observed richness)	Estimator	Average estimate	Bias	Sample s.e.	Average estimated s.e.	Sample RMSE	95% CI coverage rate
1,000 (439.7)	${\hat {S}}_{\mathrm{Chao1}}$	809.2	−190.8	65	63.8	201.6	0.3
	${\hat {S}}_{\mathrm{Jack1}}$	694.1	−305.9	23.8	24.5	306.8	0
	${\hat {S}}_{\mathrm{CB}}$	6,701.4	5,701	42,271	14,418,927	42,612	0.99
	${\hat {S}}_{\mathrm{LB}}$	1,123.1	123.1	291.8	281.3	316.4	0.96
	${\hat {S}}_{\mathrm{GP}}$	953.6	−46.4^a^	121	121	129.5^a^	0.95^a^
2,000 (629.7)	${\hat {S}}_{\mathrm{Chao1}}$	919.4	−80.6	46.1	45.3	92.8	0.64
	${\hat {S}}_{\mathrm{Jack1}}$	915.3	−84.7	24.2	23.9	88.1^a^	0.07
	${\hat {S}}_{\mathrm{CB}}$	1,612.1	612.1	328.1	305.8	694.4	0.33
	${\hat {S}}_{\mathrm{LB}}$	1,063.6	63.6	152.8	145.6	165.4	0.96^a^
	${\hat {S}}_{\mathrm{GP}}$	997.2	−2.8^a^	89.1	88.1	89.1	0.93
4,000 (817.1)	${\hat {S}}_{\mathrm{Chao1}}$	977.4	−22.6	28.5	26.9	36.3^a^	0.88
	${\hat {S}}_{\mathrm{Jack1}}$	1,059	59	21.1	19.8	62.7	0.16
	${\hat {S}}_{\mathrm{CB}}$	1,089	89	45.1	44.7	99.7	0.51
	${\hat {S}}_{\mathrm{LB}}$	1,020.8	20.8	68.8	64.2	71.8	0.94^a^
	${\hat {S}}_{\mathrm{GP}}$	1,001.7	1.7^a^	49.5	47.2	49.5	0.93
8,000 (946.5)	${\hat {S}}_{\mathrm{Chao1}}$	997.3	−2.7	12.3	12.3	12.5^a^	0.96
	${\hat {S}}_{\mathrm{Jack1}}$	1,070.9	70.9	12.9	13.1	72.1	0
	${\hat {S}}_{\mathrm{CB}}$	1,007.5	7.5	11.3	12.1	13.6	0.94
	${\hat {S}}_{\mathrm{LB}}$	1,006.0	6	23.6	23.5	24.3	0.95^a^
	${\hat {S}}_{\mathrm{GP}}$	1,000.9	0.9^a^	18.3	18.6	18.3	0.95^a^

**Notes.**

a denotes the smallest bias, smallest RMSE, and figure closest to 95% coverage.

**Table 7:** Comparison of five richness estimators based on 1,000 simulation data sets under a power decay model *p*_*i*_ ∼ 1/*i*^0.9^ with *S* = 1,000 and CV = 4. See Table 1 for the notations of the estimators.

Size *n* (Observed richness)	Estimator	Average estimate	Bias	Sample s.e.	Average estimated s.e.	Sample RMSE	95% CI coverage rate
1,000 (388.5)	${\hat {S}}_{\mathrm{Chao1}}$	786	−214	75.5	72.1	226.9	0.31
	${\hat {S}}_{\mathrm{Jack1}}$	629.2	−370.8	23.6	24.5	371.6	0
	${\hat {S}}_{\mathrm{CB}}$	2,127.4	1,127	13,983	261,663.4	14,014	0.8
	${\hat {S}}_{\mathrm{LB}}$	1,171.9	171.9	381.1	351.9	417.7	0.94^a^
	${\hat {S}}_{\mathrm{GP}}$	944.3	−55.7^a^	139.8	134.8	150.3^a^	0.94^a^
2,000 (574.1)	${\hat {S}}_{\mathrm{Chao1}}$	900.3	−99.7	52.2	51.4	112.5	0.58
	${\hat {S}}_{\mathrm{Jack1}}$	861.9	−138.1	24.7	24.7	140.3	0
	${\hat {S}}_{\mathrm{CB}}$	2,529.5	1,529	2,294.9	3,729.1	2,756	1
	${\hat {S}}_{\mathrm{LB}}$	1,074.8	74.8	178.3	175.1	193.2	0.95^a^
	${\hat {S}}_{\mathrm{GP}}$	996.7	−3.3^a^	102.6	101.8	102.7^a^	0.9
4,000 (771.6)	${\hat {S}}_{\mathrm{Chao1}}$	971.2	−28.8	31.9	31.8	42.9^a^	0.86
	${\hat {S}}_{\mathrm{Jack1}}$	1,039.9	39.9	22	21.5	45.5	0.52
	${\hat {S}}_{\mathrm{CB}}$	1,159.2	159.2	68.6	68.4	173.3	0.29
	${\hat {S}}_{\mathrm{LB}}$	1,030.5	30.5	79.6	80.5	85.2	0.96^a^
	${\hat {S}}_{\mathrm{GP}}$	1,002.7	2.7^a^	56.3	58	56.4	0.9
8,000 (923.1)	${\hat {S}}_{\mathrm{Chao1}}$	994.2	−5.8	15.2	15.2	16.3^a^	0.95^a^
	${\hat {S}}_{\mathrm{Jack1}}$	1,080.5	80.5	14.6	14.9	81.8	0
	${\hat {S}}_{\mathrm{CB}}$	1,015.3	15.3	15.3	16.2	21.6	0.89
	${\hat {S}}_{\mathrm{LB}}$	1,005.3	5.3	30.7	29.4	31.1	0.95^a^
	${\hat {S}}_{\mathrm{GP}}$	1,000.6	0.6^a^	23.6	23.1	23.6	0.93

**Notes.**

a denotes the smallest bias, smallest RMSE, and figure closest to 95% coverage.

**Table 8:** Three real datasets are used as true assemblages for simulation study, that including vascular plant species in the southern Appalachians (Miller & Wiegert, 1989), butterfly survey data in the Malayan (Fisher, Corbet & Williams, 1943), and ground-dwelling invertebrate species collected from northwest Tasmania (Bonham, Mesibov & Bashford, 2002). The first 15 frequency counts *f*_*k*_ are shown in the table for each data set.

Vascular plant species frequency counts															
*k*	1	2	3	4	5	6	7	8	9	10	11	12	13	14	≥15
*f*_*k*_	61	35	18	12	15	4	8	4	5	5	1	2	1	2	15
Butterfly species frequency counts															
*k*	1	2	3	4	5	6	7	8	9	10	11	12	13	14	≥15
*f*_*k*_	118	74	44	24	29	22	20	19	20	15	12	14	6	12	191
Ground-dwelling invertebrate species frequency counts															
*k*	1	2	3	4	5	6	7	8	9	10	11	12	13	14	≥15
*f*_*k*_	15	8	5	3	5	5	3	3	4	2	3	1	3	1	24

#### Using data sets as true assemblages

Three large biological survey data sets were used as the true assemblages and separate data sets were generated from these three assemblages. For each data set, the observed species relative abundance was treated as the true species relative abundance, and a sample of size *n* was generated through sampling with replacement. The average bias and RMSE obtained using the 1,000 generated data sets as a function of sample size are illustrated in the figures to evaluate the statistical behavior of the discussed richness estimators.

**Figure 1:**
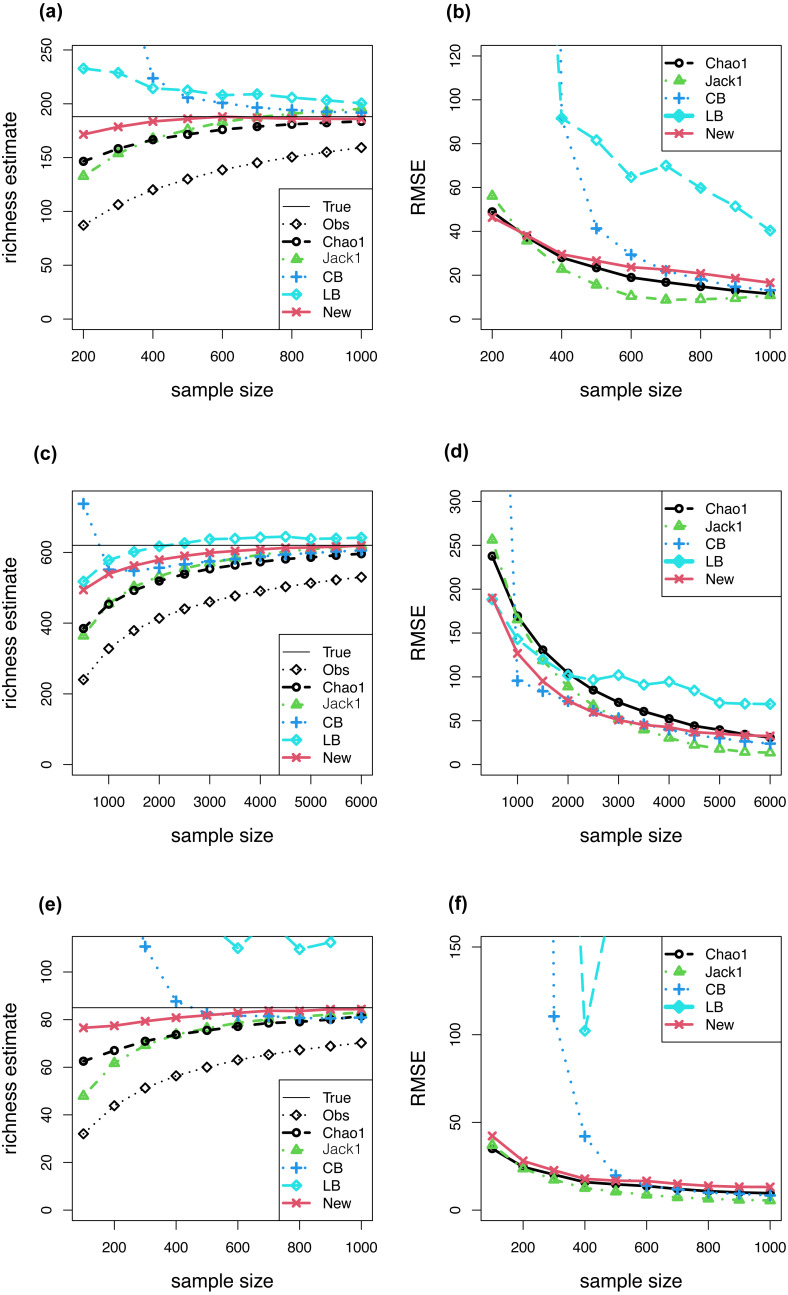
(A–F) Show the bias and RMSE of five discussed estimators as the function of sample size.

The first data set includes vascular plant species from the central portion of the Southern Appalachian region (Miller & Wiegert, 1989). This data set has a total of 188 species with 1,008 individuals; the species frequency count is presented in Table 8, and the corresponding degree of heterogeneity is presented (CV = 1.562). For each discussed estimator, the patterns of bias and RMSE as a function of sample size (from 200 to 1,000) are displayed in Figs. 1A and 1B.

The second data set comprises butterfly survey data collected from Malaya (Fisher, Corbet & Williams, 1943) and contains a total of 620 species with 9,031 individuals. The species frequency count is presented in Table 8, and the corresponding degree of heterogeneity is presented (CV = 1.435). The simulation settings were the same as those used in the abundance models. The patterns for the average bias and average RMSE as a function of sample size (from 500 to 6,000) are displayed in Figs. 1C and 1D.

The third data set contains data on ground-dwelling invertebrate species collected from northwest Tasmania (Bonham, Mesibov & Bashford, 2002) and has a total of 84 species with 2,050 individuals. The species frequency count is presented in Table 8, and the corresponding degree of heterogeneity is presented (CV = 2.07). The patterns for the average bias and average RMSE as a function of sample size (from 100 to 1,000) are illustrated in Figs. 1E and 1F.

## Discussion

In general, a good estimator should be designed with functions such that their bias and accuracy (quantified by RMSE), the two most essential properties for an estimator, decrease as the sample size increases. Furthermore, the coverage rate of the 95% confidence interval should tend towards 0.95 as the sample size increases. Another required property of a richness estimator is that the estimator should be nearly unbiased in the homogeneous model. Since it is impossible to fit all ecological communities by using a single statistical model, there is no existing uniformly unbiased richness estimator for all ecological communities. Therefore, developing a more robust estimator is the most essential goal in species richness estimation. Furthermore, based on the Cauchy–Schwarz inequality, we have the inequality of undetected richness $E \left[ {f}_{0} \right] \geq E{ \left[ {f}_{1} \right] }^{2}/2E[{f}_{2}]$, which is the essential property of undetected richness for all random samples, and the equation holds when the community is homogeneous. Therefore, the richness estimator should be an approximately unbiased estimator when species composition or species detection rate follows a homogeneous model (*i.e.,* the simplest model with only one parameter). That is why most commonly used nonparametric robust richness estimators were derived on the basis of this framework (Chao, 1984; Chao, 1987; Chao & Lee, 1992; Lee & Chao, 1994), and most parametric assumed models also include the homogeneous model as a special case, despite the fact that the homogeneous model is very rare in practice.

On the basis of these essential criteria, the following conclusions can be drawn according to the simulation results. For all simulation cases, the observed richness in the sample substantially underestimates the true richness, especially when the sample size is small or the assemblage is highly heterogeneous (see Tables 1–7).

The jackknife estimator typically results in underestimation when the sample size is small and overestimation when the sample size is large. Consequently, the jackknife estimator is unbiased in a limited range of sample sizes (see Tables 1–7). Additionally, the jackknife estimator does not meet the fundamental requirements that the bias, RMSE, and coverage rates of the 95% confidence interval should perform better as the sample size increases. In some simulation scenarios, compared to the other estimators, the jackknife estimator has the lowest RMSE due to its lower variance; however, its bias and coverage rates do not improve as the sample size increases. Although only the first-order jackknife was discussed in the manuscript, the widely used second-order jackknife estimator has similar statistical behaviors as the first-order jackknife estimator (see Chiu et al. (2014) for detail).

Since Chao1 was developed as a lower bound estimator of richness, it underestimates the true richness in most models (see Tables 2–7), especially in those with high heterogeneity. Nevertheless, Chao1 is nearly unbiased in the homogeneous model (Table 1), and its bias and RMSE decrease as the sample size increases in all discussed models (shown in Tables and Figures). Accordingly, the Chao1 estimator has the fundamental characteristics of a valuable species richness estimator. However, the estimator’s coverage rate of the 95% confidence interval derived by log-normal transformation (Chao, 1984) is generally much lower than 0.95, particularly when the sample size is small or the species composition is highly heterogeneous (Tables 3–7).

The Chao-Bunge parametric richness estimator (${\hat {S}}_{CB}$) is unreliable in sparse samples, resulting in overestimation and high variation when the sample size is small or in the cases where assemblages have high heterogeneity (shown in Tables and Figures). When the sample size is small, the Chao-Bunge estimator provides severely overestimated estimates in some cases, causing an overall increase in the average estimate. However, the Chao-Bunge estimator performs well when the sample size is large enough, which is consistent with the conclusion that “a sufficiently high overlap fraction is required to produce a reliable estimate of the species richness” (Chao & Bunge, 2002). Basically, it is an approximately unbiased estimator when a homogeneous model is assumed, and the bias and RMSE decrease as sample size increases.

The parametric estimator ${\hat {S}}_{LB}$ has been shown to be an unbiased estimator in the homogeneous model (Lanumteang & Böhning, 2011). Although the absolute value of bias and RMSE decrease as sample size increases in all discussed models, the simulation results present an inconsistent pattern in that ${\hat {S}}_{LB}$ has negative bias in the models with low heterogeneity (Tables 2–4) and positive bias in the highly heterogeneous models (Tables 5–6). When the model assumption is met (*i.e.,* a negative binomial distribution or homogeneous model), ${\hat {S}}_{LB}$ has good performance in terms of bias (Tables 1 and 7). However, like the parametric estimator ${\hat {S}}_{CB}$, ${\hat {S}}_{LB}$ usually presents an unstable estimate when the sample size is small and the assemblage is highly heterogenous (Tables 5–6).

Compared to the Chao1 estimator, the bias, RMSE, and coverage rate of the 95% confidence interval improve more for the newly proposed richness estimator as the sample size increases (Tables 1–7). The proposed estimation method provides a nearly unbiased estimator in the homogeneous model (see Table 1; also proved in Appendix S2B) and a lower bound estimator in the other discussed models (Tables 2–7). The new estimator presents a consistent pattern in all simulation cases in that the mean of the estimate is always lower than the true richness and tends to the true richness as sample size increases. Compared to the other two discussed parametric estimators (${\hat {S}}_{CB}$ and ${\hat {S}}_{LB}$), the new parametric approach presents a more stable estimate especially at small sample sizes (Tables 5–6). In most cases, the new estimator presents a higher variance, lower bias and a more accurate 95% confidence interval than the other two discussed non-parametric estimators (${\hat {S}}_{Chao1}$ and ${\hat {S}}_{Jack1}$). When the survey data are treated as true assemblages, the simulation results are consistent with those in the seven hypothetical models, and the new estimator has less bias and lower RMSE in most cases compared to the other discussed estimators (Fig. 1).

It is worth noting that although ${\hat {S}}_{LB}$ and ${\hat {S}}_{GP}$ both are derived based on the Gamma-Poisson mixture model assumption by the moment estimating method and use the number of singletons, doubletons, and tripletons to estimate undetected richness, the newly proposed estimator provides a lower but more stable estimate (*i.e.,* lower RMSE) especially at small sample size in highly heterogeneous assemblages (Tables 5–6), and provides a more accurate 95% confidence interval in most simulation models.

On the basis of the asymptotic approach, the estimated s.e.s for the discussed estimators perform well in most simulation scenarios, except for the estimate for the Chao–Bunge estimator in small sample sizes.

## Conclusions

In the literature, a plethora of approaches have been proposed for estimating total species richness in a target area. These approaches are classified as parametric or nonparametric estimators. Parametric estimators employ distribution assumptions on species compositions, and computationally expensive calculation procedures are required to solve the likelihood functions. Moreover, parametric estimators frequently fail to achieve convergence during iterative numerical procedures or result in high variance at small sample sizes. Therefore, parametric estimators are not suitable for small sample sizes and are less frequently employed in ecological studies. Conversely, nonparametric estimators with simple closed formulas and no assumptions on species composition are more robust in most simulation cases and are thus widely used in ecological studies. However, nonparametric estimators substantially underestimate total species richness when the sample size is small or when the species composition has a high degree of heterogeneity, resulting in a low coverage rate of the 95% confidence interval.

Accordingly, a new species richness estimator was proposed in this study based on the Gamma-Poisson mixture model that takes the species detection rate as a random variable to reduce the number of parameters. According to the concept of the Good–Turing frequency formula, rare species in a sample contain most of the information about undetected species. In contrast to the traditional maximum likelihood approach, the new estimator uses a simple moment method to estimate unseen richness based on observed rare species. The newly proposed estimator can also be directly derived by correcting the bias of Chao1 based on the Good-Turing frequency formula without any model assumptions. Similar to nonparametric approaches, the proposed estimator uses only the numbers of singletons, doubletons, and tripletons to estimate the number of undetected species in the sample. Compared with other widely used estimators, simulation results reveal that the proposed estimator has less bias and a lower RMSE in highly heterogenous assemblages. The asymptotic-approach-based estimator of the proposed estimator’s variance performs well in all simulation scenarios.

Overall, the newly proposed estimator uses a simplified formula and is thus more computationally efficient than other parametric approaches. In addition, the newly proposed estimator retains the flexibility of stochastic models and eliminates the divergence problem encountered in other parametric estimators. However, even though the newly proposed estimator performed well in the seven artificial models and three real data sets, it must be applied to more real data sets in the future to further demonstrate its value.

##  Supplemental Information

10.7717/peerj.14540/supp-1 Appendix S1A Newly proposed estimator is a bias-corrected Chao1 estimator

10.7717/peerj.14540/supp-2 Appendix S1B Newly proposed estimator is nearly unbiased estimator in homogeneous model

10.7717/peerj.14540/supp-3 Supplemental Information 1 R code for Tables1-7 and Figure1

## Additional Information and Declarations

### Data Availability

The following information was supplied regarding data availability: The three datasets are available from three published articles: - Miller & Wiegert (1989), DOI: https://doi.org/10.2307/1938408: https://esajournals.onlinelibrary.wiley.com/doi/abs/10.2307/1938408. - Fisher, Corbet & Williams (1943), DOI: https://doi.org/10.2307/1411: https://www.jstor.org/stable/1411?seq=1#metadata_info_tab_contents. - Bonham, Mesibov & Bashford (2002), DOI: https://doi.org/10.1016/S0378-1127(00)00717-9: https://www.sciencedirect.com/science/article/pii/S0378112700007179.
