# Supplementary material for: A more reliable species richness estimator based on the Gamma–Poisson model
Source: PeerJ. 2023 Jan 6;11:e14540. doi: 10.7717/peerj.14540 (PMC9828287; doi:10.7717/peerj.14540)
Supplement: Appendix S1A [file peerj-11-14540-s001.docx]

Supporting Information (Appendix A)

A more reliable Species Richness estimator based on Gamma–Poisson Model

Chun-Huo Chiu

[chchiu2017@ntu.edu.tw](mailto:chchiu2017@ntu.edu.tw)

Department of Agronomy, National Taiwan University

**Appendix A: Show that the newly proposed estimator is a bias-corrected estimator of Chao1 lower bound.**

Assume there are $S$ species in the target area, and $p_{i}$ is the relative abundance of species $i, i=1, 2, \ldots,S$. When a sample with size $n$ is randomly sampled from the target area, then the abundance of species in the sample $(X_{1}, X_{2},\ldots,X_{S})$ follows a multinomial distribution with parameters $n$ and $(p_{1},p_{2},\ldots,p_{S})$ and the marginal distribution of $X_{i}$ is the binomial distribution with parameters n and $p_{i}$.

Let the frequency count $f_{k}$ be the number of species that exactly detected k times in the sample, then $f_{k}$ can be formulated as $f_{k}= \sum_{i=1}^{S} I(X_{i}=k)$, where $I\left( A \right)$ is an indicator function, $I\left( A \right)$equals to 1 if $A$ occur, and 0 otherwise. Therefore, $f_{0}$ is the undetected richness in the sample, and $f_{1},$ $f_{2}$, $f_{3}$ separately are the numbers of singletons, doubletons and tripletons species.

Next, let $p_{\left( k \right)}=\sum_{i=1}^{S} p_{i}I\left( X_{i}=k \right)/f_{k}$ is the true mean relative abundance of species that exactly observed *k* times in the sample. According to the Good-Turing frequency formula (Good & Toulmin, 1956), $p_{\left( k \right)}$ can be estimated as $\hat{p}_{\left( k \right)}=\frac{\left( k+1 \right)f_{k}}{nf_{k}}, k=1, 2, \ldots$

Then, by Cauchy-Schwarz inequality, the expectation of unseen richness has the lower bound shown as

$$\sum_{i=1}^{S} \left( 1-p_{i} \right)^{n}\sum_{i=1}^{S} p_{i}^{2}\left( 1-p_{i} \right)^{n-2}\geq\left[ \sum_{i=1}^{S} p_{i}\left( 1-p_{i} \right)^{n-1} \right]^{2}$$

$$E[f_{0}]E\left[ {\frac{2}{n\left( n-1 \right)}f}_{2} \right]\geq\frac{1}{n^{2}}E\left( f_{1} \right)^{2}$$

$$E\left[ f_{0} \right]\geq\frac{n-1}{n}\frac{E\left[ f_{1} \right]^{2}}{2E[f_{2}]}$$

Since when $n$ is large enough, $\frac{n-1}{n}\approx1$. Therefore, the lower bound estimator of unseen richness can be obtained as $\frac{n-1}{n}\frac{f_{1}^{2}}{2f_{2}}\approx\frac{f_{1}^{2}}{2f_{2}}$, that also be the Chao1 estimator (Chao, 1984).

The bias of $\frac{n-1}{n}\frac{f_{1}^{2}}{2f_{2}}$ is $\frac{E\left[ \frac{1}{n}f_{1} \right]^{2}}{E[\frac{2}{n(n-1)}f_{2}]}- E\left[ f_{0} \right]$

Based on the Good-Turing frequency formula, we have following approximate equations.

$$E\left[ \frac{1}{n}f_{1} \right]=\sum_{i=1}^{S} p_{i}\left( 1-p_{i} \right)^{n-1}=\sum_{i=1}^{S} \frac{1-p_{i}}{p_{i}}{p_{i}}^{2}\left( 1-p_{i} \right)^{n-2}=\frac{2}{n(n-1)}\sum_{i=1}^{S} \frac{1-p_{i}}{p_{i}}E[I(X_{i}=2)]\approx\frac{2}{n\left( n-1 \right)}\frac{1-p_{\left( 2 \right)}}{p_{\left( 2 \right)}}E[f_{2}]$$

And

$$E\left[ f_{0} \right]=\sum_{i=1}^{S} \left( 1-p_{i} \right)^{n}=\sum_{i=1}^{S} \frac{1-p_{i}}{p_{i}}{p_{i}}\left( 1-p_{i} \right)^{n-1}=\frac{1}{n}\sum_{i=1}^{S} \frac{1-p_{i}}{p_{i}}E[I(X_{i}=1)]\approx\frac{1}{n}\frac{1-p_{(1)}}{p_{(1)}}E[f_{1}]$$

Then, the bias of Chao1 can be obtained as

$$\frac{E\left[ \frac{1}{n}f_{1} \right]^{2}}{E[\frac{2}{n(n-1)}f_{2}]}- E\left[ f_{0} \right]\approx\frac{E{\left[ \frac{1}{n}f_{1} \right]\left[ \frac{2}{n\left( n-1 \right)}\frac{1-p_{\left( 2 \right)}}{p_{\left( 2 \right)}}E\left[ f_{2} \right] \right]}}{E\left[ \frac{2}{n\left( n-1 \right)}f_{2} \right]}-\frac{1}{n}\frac{1-p_{(1)}}{p_{(1)}}E[f_{1}]$$

$$\approx\left( \frac{1-p_{\left( 2 \right)}}{p_{\left( 2 \right)}}-\frac{1-p_{(1)}}{p_{(1)}} \right)E\left[ \frac{1}{n}f_{1} \right]$$

Therefore, the bias of $\frac{n-1}{n}\frac{f_{1}^{2}}{2f_{2}}$ could be estimated by

$$\frac{1}{n}f_{1}\left( \frac{1-\hat{p}_{\left( 2 \right)}}{\hat{p}_{\left( 2 \right)}}-\frac{1-\hat{p}_{(1)}}{\hat{p}_{(1)}} \right),$$

where $\hat{p}_{\left( 2 \right)}=\frac{3f_{3}}{tn}$ and $\hat{p}_{\left( 1 \right)}=\frac{2f_{2}}{nf_{1}}$. When $n$ is large enough, we have the approximation, type

$$\frac{1}{n}f_{1}\left( \frac{1-\hat{p}_{\left( 2 \right)}}{\hat{p}_{\left( 2 \right)}}-\frac{1-\hat{p}_{(1)}}{\hat{p}_{(1)}} \right)\approx\frac{f_{1}^{2}}{2f_{2}}\left( \frac{2f_{2}^{2}}{3f_{1}f_{3}}-1 \right)$$

Therefore, the bias-corrected estimator of Chao1 lower bound can be obtained as

$$\frac{n-1}{n}\frac{f_{1}^{2}}{2f_{2}}+\frac{n-1}{n}\frac{f_{1}^{2}}{2f_{2}}\left( 1-\frac{2f_{2}^{2}}{3f_{1}f_{3}} \right)\approx\frac{f_{1}^{2}}{2f_{2}}\left( 2-\frac{2f_{2}^{2}}{3f_{1}f_{3}} \right)$$

that is identical to the proposed estimator in the main text and ends the proof.

References:

Chao, A. (1984). Nonparametric estimation of the number of classes in a population. *Scandinavian Journal of statistics*, 265-270.

Good, I. J., and G. Toulmin. 1956. The Number of New Species and the Increase of Population Coverage When a Sample Is Increased. *Biometrika* 43:45-63.
