# Supplementary material for: A more reliable species richness estimator based on the Gamma–Poisson model
Source: PeerJ. 2023 Jan 6;11:e14540. doi: 10.7717/peerj.14540 (PMC9828287; doi:10.7717/peerj.14540)
Supplement: Appendix S1B [file peerj-11-14540-s002.docx]

Supporting Information (Appendix B)

A more reliable Species Richness estimator based on Gamma–Poisson Model

Chun-Huo Chiu

[chchiu2017@ntu.edu.tw](mailto:chchiu2017@ntu.edu.tw)

Department of Agronomy, National Taiwan University

**Appendix B: Show that the proposed estimator is nearly unbiased estimator under homogeneous model.**

Under homogeneous model $p_{i}=p=\frac{1}{S}$, $\forall j=1, 2,\ldots,S.$ the new proposed estimator is unbiased estimator of species richness, i.e. $\lim_{S\to\infty} \frac{{E[\hat{S}}_{GP}]}{S}=1$

Proof:

Assume there are $S$ species in the target area, and $p_{i}$ is the relative abundance of species $i, i=1, 2, \ldots,S$. When a sample with size $n$ is randomly sampled from the target area, then the abundance of species in the sample $(X_{1}, X_{2},\ldots,X_{S})$ follows a multinomial distribution with parameters $n$ and $(p_{1},p_{2},\ldots,p_{S})$ and the marginal distribution of $X_{i}$ is the binomial distribution with parameters n and $p_{i}$.

Let the frequency count $f_{k}$ be the number of species that exactly detected k times in the sample, then $f_{k}$ can be formulated as $f_{k}= \sum_{i=1}^{S} I(X_{i}=k)$, where $I\left( A \right)$ is an indicator function, $I\left( A \right)$equals to 1 if $A$ occur, and 0 otherwise. Therefore, $f_{0}$ is the undetected richness in the sample, and $f_{1},$ $f_{2}$, $f_{3}$ separately are the numbers of singletons, doubletons and tripletons species.

Then, under homogeneous model, we have following equations:

1. $E\left[ f_{0} \right]=SP\left( X_{i}=0 \right)=S\left( 1-p \right)^{T}$
2. $E\left[ f_{1} \right]=SP\left( X_{i}=1 \right)=Snp\left( 1-p \right)^{n-1}$
3. $E\left[ f_{2} \right]=SP\left( X_{i}=2 \right)=S\frac{n\left( n-1 \right)}{2!}p^{2}\left( 1-p \right)^{n-2}$
4. $E\left[ f_{3} \right]=SP\left( X_{i}=3 \right)= S\frac{n(n-1)(n-2)}{3!}p^{3}\left( 1-p \right)^{n-3}$
5. $E\left[ S_{obs} \right]=SP\left( X_{i}>0 \right)=S (1-\left( 1-p \right)^{n})$

The new proposed estimator shown as

$$\hat{S}_{GP}=S_{obs}+\frac{f_{1}^{2}}{2f_{2}}\left( 2-\frac{2f_{2}^{2}}{3f_{1}f_{3}} \right)=S_{obs}+(\frac{f_{1}^{2}}{f_{2}}-\frac{f_{1}f_{2}}{3f_{3}})$$

Since, when $n$ is large enough, we have following two approximate equations.

$$E\left[ \frac{f_{1}^{2}}{f_{2}} \right]=\frac{\left[ Snp\left( 1-p \right)^{n-1} \right]^{2}}{S\frac{n\left( n-1 \right)}{2!}p^{2}\left( 1-p \right)^{n-2}}=2\frac{n}{n-1}S\left( 1-p \right)^{n}\approx2S\left( 1-p \right)^{n},$$

and

$$E\left[ \frac{f_{1}f_{2}}{3f_{3}} \right]=\frac{Snp\left( 1-p \right)^{n-1}S\times\frac{n\left( n-1 \right)}{2!}p^{2}\left( 1-p \right)^{n-2}}{3S\frac{n(n-1)(n-2)}{3!}p^{3}\left( 1-p \right)^{n-3}}=\frac{n}{n-2}S\left( 1-p \right)^{n}\approx S\left( 1-p \right)^{n}.$$

Therefore, when *n* is large enough, $\frac{E[\frac{f_{1}^{2}}{f_{2}}-\frac{f_{1}f_{2}}{3f_{3}}]}{S}$ converge to $2\left( 1-p \right)^{n}-\left( 1-p \right)^{n}=\left( 1-p \right)^{n}$

Then, $\frac{E\left[ \hat{S}_{GP} \right]}{S}=\frac{E\left[ S_{obs} \right]}{S}+\frac{E[\frac{f_{1}^{2}}{f_{2}}-\frac{f_{1}f_{2}}{3f_{3}}]}{S}$converges to $(1-\left( 1-p \right)^{n})+\left( 1-p \right)^{n}=1$ and ends the proof.
